# Evaluating the efficiency of a nomogram based on the data of neurosurgical intensive care unit patients to predict pulmonary infection of multidrug-resistant *Acinetobacter baumannii*

**DOI:** 10.3389/fcimb.2023.1152512

**Published:** 2023-04-25

**Authors:** Di Wu, Zhuang Sha, Yibing Fan, Jiangyuan Yuan, Weiwei Jiang, Mingqi Liu, Meng Nie, Chenrui Wu, Tao Liu, Yupeng Chen, Jiancheng Feng, Shiying Dong, Jin Li, Jian Sun, Chongjie Pang, Rongcai Jiang

**Affiliations:** ^1^ Department of Neurosurgery, Tianjin Medical University General Hospital, Tianjin, China; ^2^ Tianjin Neurological Institute, Key Laboratory of Post Neuro-Injury Neuro-Repair and Regeneration in Central Nervous System, Ministry of Education, Tianjin, China; ^3^ Department of Clinical Laboratory, Tianjin Medical University General Hospital, Tianjin, China; ^4^ Department of Infectious Diseases, Tianjin Medical University General Hospital, Tianjin, China

**Keywords:** *Acinetobacter baumannii*, pulmonary infection, multidrug-resistant, nomogram, early diagnosis, neurosurgical intensive care unit

## Abstract

**Background:**

Pulmonary infection caused by multidrug-resistant *Acinetobacter baumannii* (MDR-AB) is a common and serious complication after brain injury. There are no definitive methods for its prediction and it is usually accompanied by a poor prognosis. This study aimed to construct and evaluate a nomogram based on patient data from the neurosurgical intensive care unit (NSICU) to predict the probability of MDR-AB pulmonary infection.

**Methods:**

In this study, we retrospectively collected patient clinical profiles, early laboratory test results, and doctors’ prescriptions (66 variables). Univariate and backward stepwise regression analyses were used to screen the variables to identify predictors, and a nomogram was built in the primary cohort based on the results of a logistic regression model. Discriminatory validity, calibration validity, and clinical utility were evaluated using validation cohort 1 based on receiver operating characteristic curves, calibration curves, and decision curve analysis (DCA). For external validation based on predictors, we prospectively collected information from patients as validation cohort 2.

**Results:**

Among 2115 patients admitted to the NSICU between December 1, 2019, and December 31, 2021, 217 were eligible for the study, including 102 patients with MDR-AB infections (102 cases) and 115 patients with other bacterial infections (115 cases). We randomly categorized the patients into the primary cohort (70%, N=152) and validation cohort 1 (30%, N=65). Validation cohort 2 consisted of 24 patients admitted to the NSICU between January 1, 2022, and March 31, 2022, whose clinical information was prospectively collected according to predictors. The nomogram, consisting of only six predictors (age, NSICU stay, Glasgow Coma Scale, meropenem, neutrophil to lymphocyte ratio, platelet to lymphocyte ratio), had significantly high sensitivity and specificity (primary cohort AUC=0.913, validation cohort 1 AUC=0.830, validation cohort 2 AUC=0.889) for early identification of infection and had great calibration (validation cohort 1,2 P=0.3801, 0.6274). DCA confirmed that the nomogram is clinically useful.

**Conclusion:**

Our nomogram could help clinicians make early predictions regarding the onset of pulmonary infection caused by MDR-AB and implement targeted interventions.

## Introduction

*Acinetobacter* spp. are key pathogens in nosocomial infections. In recent decades, *Acinetobacter baumannii* (AB) infection has become a global clinical challenge. Carbapenems are the treatment of choice for *Acinetobacter* infections, especially in critically ill patients (Chakravarty, 2020). With the advent of the multidrug resistance era, the prevalence of carbapenem-resistant AB has increased. Consequently, the World Health Organization (WHO) has ranked it as the world’s top pathogen, urgently requiring novel antibiotics (World Health Organization, 2017). The emergence of multidrug-resistant *Acinetobacter baumannii* (MDR-AB) poses greater challenges for clinical treatment. Almost all of these strains are resistant to commonly used antibiotics to varying degrees; hence, infections caused by such strains are difficult to eradicate (Ibrahim et al., 2021). Currently, MDR-AB can cause pulmonary, bloodstream, central nervous system, urinary tract, and abdominal infections; the most common of these are pulmonary infections (Kengkla et al., 2017).

Recent data show that the overall prevalence of multidrug resistant strains in patients with hospital-acquired pneumonia (HAP) and ventilator-associated pneumonia (VAP) caused by AB is approximately 79.9%, and the overall mortality rate can be as high as 56.2% (Lim et al., 2019). The annual incidence of pneumonia caused by MDR-AB in intensive care units (ICU) is increasing. Effectively controlling its incidence has attracted extensive attention from medical institutions (Russo et al., 2021).

Nomograms are predictive tools that create simple graphical representations of statistical predictive models and are used to generate numerical probabilities of clinical events (Cahlon et al., 2012). In recent years, studies using nomograms to predict the occurrence and prognosis of diseases have emerged. Nomograms have been constructed to predict pulmonary, bloodstream, urinary tract, and surgical wound infections (Tien et al., 2018; Chen et al., 2022; Yan et al., 2022). Zhang et al. conducted a retrospective study in which they developed a nomogram to predict the risk of acquiring carbapenem-resistant microorganism infections in patients admitted to the ICU for the first time (Zhang et al., 2022). With the coronavirus disease 2019 (COVID-19) affecting the world, Gong et al. constructed a nomogram for early identification of patients at risk of severe COVID-19 (Gong et al., 2020). However, nomograms predicting the risk of pulmonary infection caused by MDR-AB have received little attention. Based on the validation of traditional risk factors, we conducted a retrospective and prospective study to identify novel risk factors and their associated effects based on neurosurgical ICU (NSICU) profiles. Specifically, we created a scale to evaluate the probability of MDR-AB pneumonia in patients undergoing critical neurosurgical care, thereby guiding clinical practice.

## Materials and methods

### Patient recruitment

**Figure 1:**
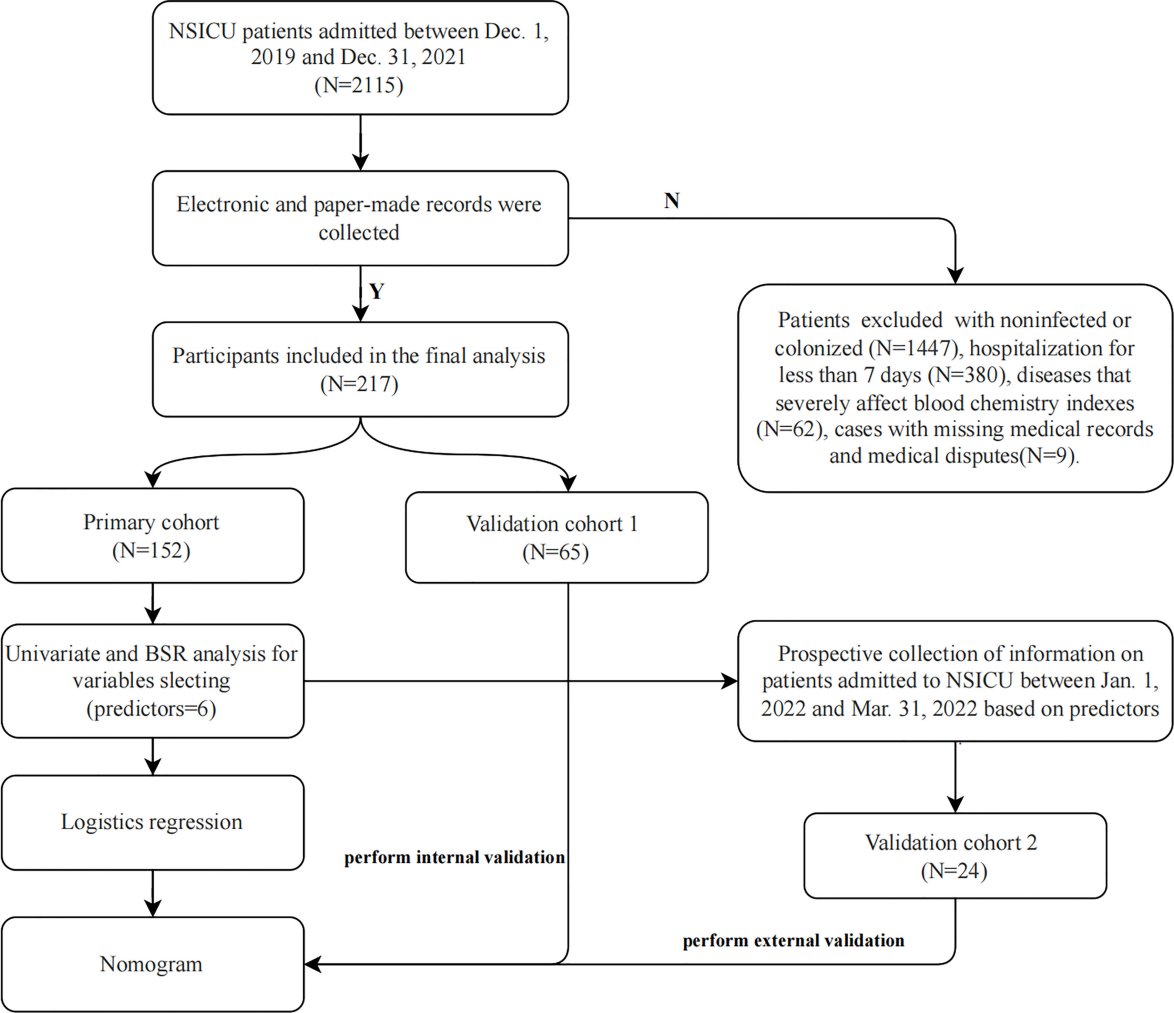
Flow chart of the study. *BSR*, backward stepwise regression.

This study included patients admitted to the NSICU of Tianjin Medical University General Hospital (the highest-level hospital located in the center of Tianjin, China) between December 1, 2019, and December 31, 2021. Patients with the following conditions were excluded: non-infected or colonized patients; hospitalization for less than 7 days; diseases that severely affect blood chemistry indexes (such as leukemia, severe renal failure, severe liver failure, etc.), and cases with missing medical records and subject to medical disputes. Next, we prospectively collected information on patients admitted to the NSICU between January 1, 2022, and March 31, 2022, based on predictors (**Figure 1**).

Based on the draft standardized international terminology for multidrug resistant (MDR), extensively-drug resistant (XDR), and pan-drug resistant (PDR) bacteria proposed by experts from the United States and other countries in 2010, Magiorakos and other experts officially published the interim standard definitions of MDR, XDR, and PDR (Magiorakos et al., 2012). The categories of antimicrobials include aminoglycosides (e.g., gentamicin, tobramycin, or amikacin), antipseudomonal carbapenems (e.g., imipenem or meropenem), antipseudomonal fluoroquinolones (e.g., ciprofloxacin or levofloxacin), antipseudomonal penicillins + β-lactamase inhibitors (e.g., piperacillin-tazobactam or ticarcillin-clavulanic acid), extended-spectrum cephalosporins (e.g., ceftazidime, ceftriaxone, or cefepime), folate pathway inhibitors (e.g., trimethoprim-sulfamethoxazole), penicillin + β-lactamase inhibitors (e.g., ampicillin-sulbactam), polymyxins (e.g., colistin or polymyxin B), and tetracyclines (e.g., tetracycline, doxycycline, or minocycline). The criteria for MDR, XDR and PDR were defined based on the non-susceptibility to the above categories. The MDR criteria for *Acinetobacter* spp. was defined as acquired non-susceptibility to at least one agent from three or more antimicrobial categories listed above. XDR was defined as non-susceptibility to at least one agent from all but two or fewer antimicrobial categories. PDR was defined as non-susceptibility to all agents in all of the above antimicrobial categories. Therefore, a bacterial isolate characterized as XDR would also be characterized as MDR. Similarly, a bacterial isolate must be XDR to be further defined as PDR (Magiorakos et al., 2012).

The patients were divided into an “MDR-AB pneumonia group” and an “other bacterial pneumonia group.” The inclusion criteria for the MDR-AB pneumonia group (diagnostic criteria) were as follows: (1) clinical symptoms and signs consistent with pneumonia (fever >38°C, increased white blood cell count to >12 × 10^9^, cough, thick sputum with pulmonary rales, dyspnea, etc.) and new, progressive, or persistent pulmonary infiltrates and/or solid lesions on imaging (Klompas et al., 2012); and (2) positive sputum culture results: two or more sputum cultures showing pure MDR-AB growth or MDR-AB dominant growth (Horan et al., 2008). The inclusion criteria for the other bacterial pneumonia group were as follows: (1) clinical symptoms, signs, and imaging changes compatible with pneumonia; and (2) sputum culture showing growth of other bacteria or dominant growth of other bacteria. The culture results included more than 15 species of bacteria, including *Neisseria*, *Klebsiella pneumoniae*, *Pseudomonas aeruginosa*, and *Staphylococcus* (*epidermidis, aureus*, and *hemolysis*), except AB. The respiratory specimens collected included endotracheal aspiration (ETA), bronchoalveolar lavage fluid (BALF), and a protected specimen brush (PSB), in addition to the patient’s sputum. They were acquired from the patient’s lower respiratory tract and were fresh, yellow, purulent, and not contaminated by the upper respiratory tract. The qualifying criteria for a respiratory specimen were <10 epithelial cells and ≥ 25 leukocytes per microscopic field (Heineman et al., 1977).

### Data collection

We retrospectively collected the patient’s data, including (1) patient demographics, such as sex, age, and underlying disease; (2) major diagnoses and comorbidities; (3) total duration of hospitalization and NSICU stay; (4) relevant scores and nursing status; (5) invasive operations; (6) ventilator usage time and whether tracheal intubation or tracheotomy was performed; and (7) application of antibiotics. For the MDR-AB pneumonia group, the type of antibiotic applied before the patient contracted MDR-AB pneumonia was recorded. For the other bacterial infection group, the type of antibiotic administered during the patient’s hospitalization was recorded; (8) early hospitalization (3–7 days after admission) blood test indicators such as white blood cell count (WBC), platelet count (PLT), absolute neutrophil count (NEU), absolute lymphocyte count (LYM), absolute monocyte count (MON), hemoglobin (HGB), globulin (GLO), albumin (ALB), alanine transaminase (ALT), aspartate transaminase (AST), creatinine (CREA), neutrophil to lymphocyte ratio (NLR), lymphocyte to monocyte ratio (LMR), platelet to lymphocyte ratio (PLR), C-reactive protein (CRP), procalcitonin (PCT), D-dimer, and glucose (GLU). This study met medical ethics standards (Declaration of Helsinki). The retrospective collection of data from the patients and their analyses was approved by the ethics committee of Tianjin Medical University General Hospital.

### Statistical analyses

We randomly categorized the patients retrospectively into the primary cohort (70%) and validation cohort 1 (30%). The data from the primary cohort were used to construct nomograms and the data from the validation cohort 1 were used for internal validation. These variables are presented as means [standard deviation (SD)] or medians [interquartile range (IQR)]. The independent samples t-test or Mann–Whitney U test was used to compare two independent groups. Categorical variables are presented as numbers (percentage), and their differences among groups were compared using the chi-square test or Fisher’s exact test. Parameters identified as significant (P<0.05) in univariate analysis were incorporated into multivariate logistic regression analysis using a backward stepwise regression (BSR) approach. Optimal model selection was performed using the likelihood-ratio test using the Akaike information criterion (AIC). The identified independent factors were analyzed again using logistic regression models, and the results were expressed as odds ratios (OR) and 95% confidence intervals (CI). Finally, we constructed a nomogram based on the results of a logistic regression model and conducted a Hosmer–Lemeshow (HL) test to evaluate the calibration. The area under the receiver operating characteristic curve (AUC) was used to assess the recognition ability of the nomogram. According to the Youden Index, receiver operating characteristic (ROC) curves were used to determine the optimal cut-off point and its sensitivity and specificity. Decision curve analysis (DCA) curves were used to assess the clinical applicability of the nomogram. In addition, we used prospectively collected patient data as validation cohort 2 to perform external validation of the nomogram we constructed. All tests were two-tailed, and a P-value <0.05 was regarded as statistically significant.

Statistical analyses were performed using the SPSS software (version 25.0), and the nomogram was constructed using the R programming language (version 4.2.0; http://www.r-project.org/). The packages in R used in this study were “MASS,” “survival,” “rms,” “rmda,” “car,” “Hmisc,” “pROC,” and “ResourceSelection.”

## Results

### Clinical characteristics

We retrospectively collected the clinical data of 2115 patients. Overall, 217 patients who met the criteria were enrolled in the study (102 in the MDR-AB pneumonia group and 115 in the other bacterial pneumonia group). Nineteen patients with XDR-AB and 0 patients with PDR-AB were included in the MDR-AB pneumonia group. Pulmonary infection prevalence caused by MDR-AB in the NSICU was as high as 4.82%, with XDR-AB pneumonia accounting for 18.6%. A total of 66 variables were collected for each patient. **Table S1** presents the clinical characteristics of the two groups. The 217 patients were randomly divided into 152 and 65 patients in the primary cohort (MDR-AB pneumonia group, N=74; other bacterial pneumonia group, N=78) and validation cohort 1 (MDR-AB pneumonia group, N=28; other bacterial pneumonia group, N=37), respectively. The clinical characteristics of the two cohorts and a comparison of the randomization assignments are presented in **Table 1**. The results showed satisfactory similarity in the prevalence of MDR-AB pneumonia in both cohorts (P = 0.542). In addition, the clinical parameters, use of antibiotics, and early hospitalization blood test indicators were comparable between the cohorts (all P > 0.05). In summary, the parameters selected for this study were homogeneous and comparable across cohorts, indicating that the clinical data were reliably sourced and collected with quality assurance.

**Table 1:** Clinical characteristics of the patients in the primary cohort and validation cohort 1.

Variables	Primary cohort			Validation cohort 1		P-value^a^
	Case group (N=74)	Control group (N=78)	P-value	Case group (N=28)	Control group (N=37)	0.542
On admission						
Gender (males)	47 (63.5)	58 (74.4)	0.148	20 (71.4)	26 (70.3)	1.000
Age (years)	61.6 ± 15.6	61.4 ± 14.0	0.939	62.2 ± 19.6	59.6 ± 16.0	0.880
Primary diagnosis						
Cerebral hemorrhage	40 (54.1)	43 (55.1)	0.894	14 (50.0)	15 (40.5)	0.230
Traumatic brain injury	26 (35.1)	18 (23.1)	0.101	14 (50.0)	8 (21.6)	0.577
Intracranial space occupying lesion	4 (5.41)	3 (3.85)	0.714	0 (0.00)	5 (13.5)	0.350
Intracranial infections	3 (4.05)	1 (1.28)	0.357	0 (0.00)	0 (0.00)	0.319
Ischemic cerebrovascular disease	5 (6.76)	7 (8.97)	0.612	0 (0.00)	4 (10.8)	0.782
Aneurysm	2 (2.70)	8 (10.3)	0.060	0 (0.00)	4 (10.8)	1.000
Others	2 (2.70)	3 (3.85)	1.000	0 (1.96)	2 (5.41)	1.000
Comorbidities						
Rib fractures	7 (9.46)	4 (5.13)	0.303	6 (21.4)	3 (8.11)	0.199
Pulmonary contusion	6 (8.10)	3 (3.85)	0.318	2 (7.14)	3 (8.11)	0.763
Systemic multiple fractures	10 (13.5)	4 (5.13)	0.074	7 (25.0)	3 (8.11)	0.275
Hypertension	40 (54.1)	47 (60.3)	0.440	15 (53.6)	25 (67.6)	0.661
Diabetes	16 (21.6)	18 (23.1)	0.830	8 (28.6)	11 (29.7)	0.365
Hyperlipidemia	4 (5.41)	6 (7.69)	0.570	2 (7.14)	2 (5.41)	1.000
Obsolete cerebral infarction	18 (24.3)	15 (19.2)	0.446	6 (21.4)	8 (21.6)	1.000
Obsolete cerebral hemorrhage	10 (13.5)	7 (8.97)	0.375	2 (7.14)	1 (2.71)	0.202
Atrial fibrillation	2 (2.70)	5 (6.41)	0.443	1 (3.57)	0 (0.00)	0.441
Coronary heart disease	9 (12.2)	11 (14.1)	0.724	3 (10.7)	6 (16.2)	1.000
After admission						
Total duration of hospitalization (days)	47.0 (31.8,68.3)	30.0 (21.0,44.0)	**<0.001**	45.0 (21.5,83.5)	35.0 (21.5,53.5)	0.952
NSICU stay (days)	30.5 (20.0,44.3)	14.0 (9.00,18.5)	**<0.001**	24.0 (19.0,42.3)	14.0 (7.50,20.0)	0.580
GCS (points)	7.00 (4.00,8.25)	11.0 (8.00,13.0)	**<0.001**	6.00 (5.00,8.75)	10.0 (9.00,12.5)	0.702
Pressure ulcer risk assessment score	12.0 (11.0,12.3)	12.0 (11.0,13.0)	0.063	12.0 (11.0,13.0)	12.0 (11.0,12.5)	0.552
APACHE II score (points)			**0.003**			0.747
10- <15	17 (22.9)	38 (48.7)		9 (32.1)	17 (46.0)	
15- <25	46 (62.2)	35 (44.9)		14 (50.0)	17 (45.9)	
≥25	11 (14.9)	5 (6.41)		5 (17.9)	3 (8.11)	
Ventilator usage time (hours)			**<0.001**			0.800
<24	21 (28.4)	50 (64.1)		6 (21.4)	22 (59.5)	
24- <96	10 (13.5)	13 (16.7)		6 (21.4)	6 (16.2)	
≥96	43 (58.1)	15 (19.2)		16 (57.1)	9 (24.3)	
Tracheal intubation	48 (64.9)	47 (60.3)	0.557	19 (67.9)	24 (64.9)	0.720
Tracheotomy	26 (35.1)	15 (19.2)	**0.027**	7 (25.0)	7 (18.9)	0.501
Electronic bronchoscopy	21 (28.4)	8 (10.3)	**0.004**	12 (42.9)	4 (10.8)	0.460
Urinary catheterization	70 (94.6)	75 (96.2)	0.647	27 (96.4)	37 (100.0)	0.441
Gastric tube placement	66 (89.2)	69 (88.5)	0.887	27 (96.4)	321 (86.5)	0.851
Tertiary/quaternary surgery	38 (51.4)	44 (56.4)	0.532	12 (42.9)	23 (62.2)	1.000
Lumbar puncture	27 (36.5)	13 (16.7)	**0.006**	9 (32.1)	10 (27.0)	0.783
Limb motor ability	36 (48.6)	46 (59.0)	0.202	12 (42.9)	20 (54.1)	0.755
Protective restraint	36 (48.6)	41 (52.6)	0.629	11 (39.3)	17 (45.9)	0.381
Antibiotic applications						
Cefazolin	5 (6.76)	3 (3.85)	0.486	1 (3.57)	0 (0.00)	0.285
Cefoxitin	32 (43.2)	39 (50.0)	0.404	14 (50.0)	15 (40.5)	0.893
Cefuroxime	1 (1.35)	5 (6.41)	0.210	1 (3.57)	2 (5.41)	1.000
Ceftriaxone	8 (10.8)	5 (6.41)	0.332	2 (7.14)	5 (13.5)	0.794
Ceftazidime	2 (2.70)	2 (2.56)	1.000	1 (3.57)	0 (0.00)	1.000
Vancomycin	21 (28.4)	13 (16.7)	0.083	5 (17.9)	13 (35.1)	0.504
Linezolid	9 (12.2)	6 (7.69)	0.356	4 (14.3)	1 (2.70)	0.801
Imipenem	8 (10.8)	9 (11.5)	0.887	4 (14.3)	3 (8.11)	1.000
Meropenem	40 (54.1)	16 (20.5)	**<0.001**	13 (46.4)	11 (29.7)	1.000
Biapenem	1 (1.35)	0 (0.00)	0.487	1 (3.57)	1 (2.70)	0.214
Piperacillin-tazobactam	39 (52.7)	50 (64.1)	0.154	23 (82.1)	24 (64.9)	0.077
Cefoperazone-sulbactam	23 (31.1)	26 (33.3)	0.767	5 (17.9)	22 (59.5)	0.246
Teicoplanin	3 (4.05)	5 (6.41)	0.720	2 (5.41)	2 (7.14)	0.755
Levofloxacin	1 (1.35)	4 (5.13)	0.368	1 (3.57)	4 (10.8)	0.170
Laboratory test						
WBC (×10^9^/L)	9.74 (8.29,11.7)	10.3 (8.56,12.2)	0.292	10.5 (9.17,12.2)	10.1 (7.68,11.4)	0.755
PLT (×10^9^/L)	161.3 (127.5,192.5)	164.0 (140.0,198.6)	0.269	156.8 (118.0,197.5)	166.0 (139.3,223.0)	0.856
NEU (×10^9^/L)	8.13 (6.67,10.1)	8.87 (6.96,10.4)	0.342	8.66 (7.50,11.0)	8.53 (6.36,9.53)	0.766
LYM (×10^9^/L)	0.67 (0.49,0.88)	0.85 (0.63,1.11)	**0.006**	0.65 (0.53,0.95)	0.81 (0.60,1.12)	0.886
MON (×10^9^/L)	0.73 (0.55,0.90)	0.76 (0.62,0.96)	0.257	0.83 (0.69,1.04)	0.72 (0.56,0.90)	0.523
HGB (g/L)	102.5 (91.0,121.6)	114.0 (96.0,129.8)	**0.029**	101.3 (83.0,115.8)	116.0 (101.0,128.3)	0.970
GLO (g/L)	29.5 (26.4,32.1)	28.5 (26.0,31.1)	0.192	29.5 (25.5,32.5)	29.5 (28.0,31.8)	0.621
ALB (g/L)	31.0 (28.5,34.0)	32.3 (30.0,35.0)	**0.044**	30.8 (27.6,34.6)	31.5 (29.3,33.5)	0.470
ALT (U/L)	31.5 (17.5,66.1)	29.8 (16.4,47.5)	0.110	31.5 (23.0,59.3)	27.5 (19.5,47.8)	0.424
AST (U/L)	46.3 (31.0,71.9)	32.8 (25.4,48.3)	**<0.001**	40.5 (28.1,52.3)	32.0 (21.3,64.5)	0.568
CREA (umol/L)	53.8 (45.0,76.0)	57.8 (47.6,69.6)	0.664	67.8 (51.4,87.9)	52.0 (42.5,71.5)	0.466
NLR	12.6 (8.09,16.4)	8.89 (6.87,15.8)	**0.047**	13.1 (9.46,21.3)	10.2 (7.22,16.6)	0.546
LMR	0.93 (0.67,1.49)	1.07 (0.81,1.37)	0.250	0.81 (0.63,1.00)	1.19 (0.76,1.57)	0.546
PLR	241.8 (188.5,313.6)	193.0 (152.2,270.8)	**0.025**	202.7 (177.5,285.8)	211.9 (142.3,301.9)	0.974
CRP (mg/dL)	7.60 (3.74,11.9)	7.70 (4.07,11.1)	0.923	7.34 (3.93,10.7)	5.94 (2.83,10.8)	0.513
PCT (ng/mL)	0.27 (0.07,0.77)	0.26 (0.05,0.69)	0.507	0.58 (0.14,1.16)	0.14 (0.05,0.84)	0.269
D-Dimer(ng/mL)	2224.5 (1056.3,5402.3)	1820.5 (800.0,3410.0)	0.115	3458.0 (1607.0,4998.0)	1277.0 (813.5,3673.5)	0.739
GLU(umol/L)	8.00 (7.00,10.0)	8.00 (6.75,10.0)	0.694	8.00 (7.00,10.8)	7.00 (5.00,9.75)	0.300

Data are shown as number (percentage), mean ± standard deviation, or median (quartile).

The p-value column is marked in bold with the 15 significantly different variables in the univariate analysis.

a P-value for the comparison of each parameter in the two cohorts.

NSICU, neurosurgical intensive care unit; GCS, Glasgow Coma Scale; WBC, white blood cell count; PLT, platelets count; NEU, absolute neutrophil count; LYM, absolute lymphocyte count; MON, absolute monocyte count; HGB, hemoglobin; GLO, globulin; ALB, albumin; ALT, alanine transaminase; AST, aspartate transaminase; NLR, neutrophil to lymphocyte ratio; LMR, lymphocyte to monocyte ratio; PLR, platelet to lymphocyte ratio; CRP, C-reactive protein; PCT, descendants; GLU, glucose.

### Variables selection using the BSR analysis

The BSR approach has a significant advantage in variable selection since it calculates all possible combinations of variables, and the final combination selected is the optimal combination based on the minimum AIC. In **Table 1**, the results of the univariate analysis show that 15 variables in the primary cohort were significantly different: total duration of hospitalization, NSICU stay, Glasgow Coma Scale (GCS) score, APACHE II score, ventilator usage time, tracheotomy, electronic bronchoscopy, lumbar puncture, meropenem, LYM, HGB, ALB, AST, NLR, and PLR (all P<0.05). We incorporated sex, age, and the 15 variables mentioned above in the BSR model. After eliminating the variables with poor predictive performance or multicollinearity, we screened six predictors. We then included them in a multivariable logistic regression, and the results showed that older age (OR=1.03; 95% CI=0.99–1.06), prolonged NSICU stay (OR=1.11; 95% CI=1.05–1.16), lower GCS score (OR=0.68; 95% CI=0.56–0.82), application of meropenem (OR=3.92; 95% CI=1.41–10.9), lower NLR (OR=0.90; 95% CI=0.83–0.98), and higher PLR (OR=1.01; 95% CI=1.00–1.02) were independent risk factors for pulmonary infections caused by MDR-AB (**Table 2**).

**Table 2:** Results of the backward stepwise regression analysis.

Predictor	β	OR	95%CI	P
Age (years)	0.025	1.03	0.99-1.06	0.118
NSICU stay (days)	0.101	1.11	1.05-1.16	<0.001
GCS (points)	-0.385	0.68	0.56-0.82	<0.001
Meropenem	1.366	3.92	1.41-10.9	0.009
NLR	-0.101	0.90	0.83-0.98	0.016
PLR	0.009	1.01	1.00-1.02	0.005

NSICU, neurosurgical intensive care unit; GCS, Glasgow Coma Scale; NLR, neutrophil to lymphocyte ratio; PLR, platelet to lymphocyte ratio.

### Construction, validation, and evaluation of the nomogram

**Figure 2:**
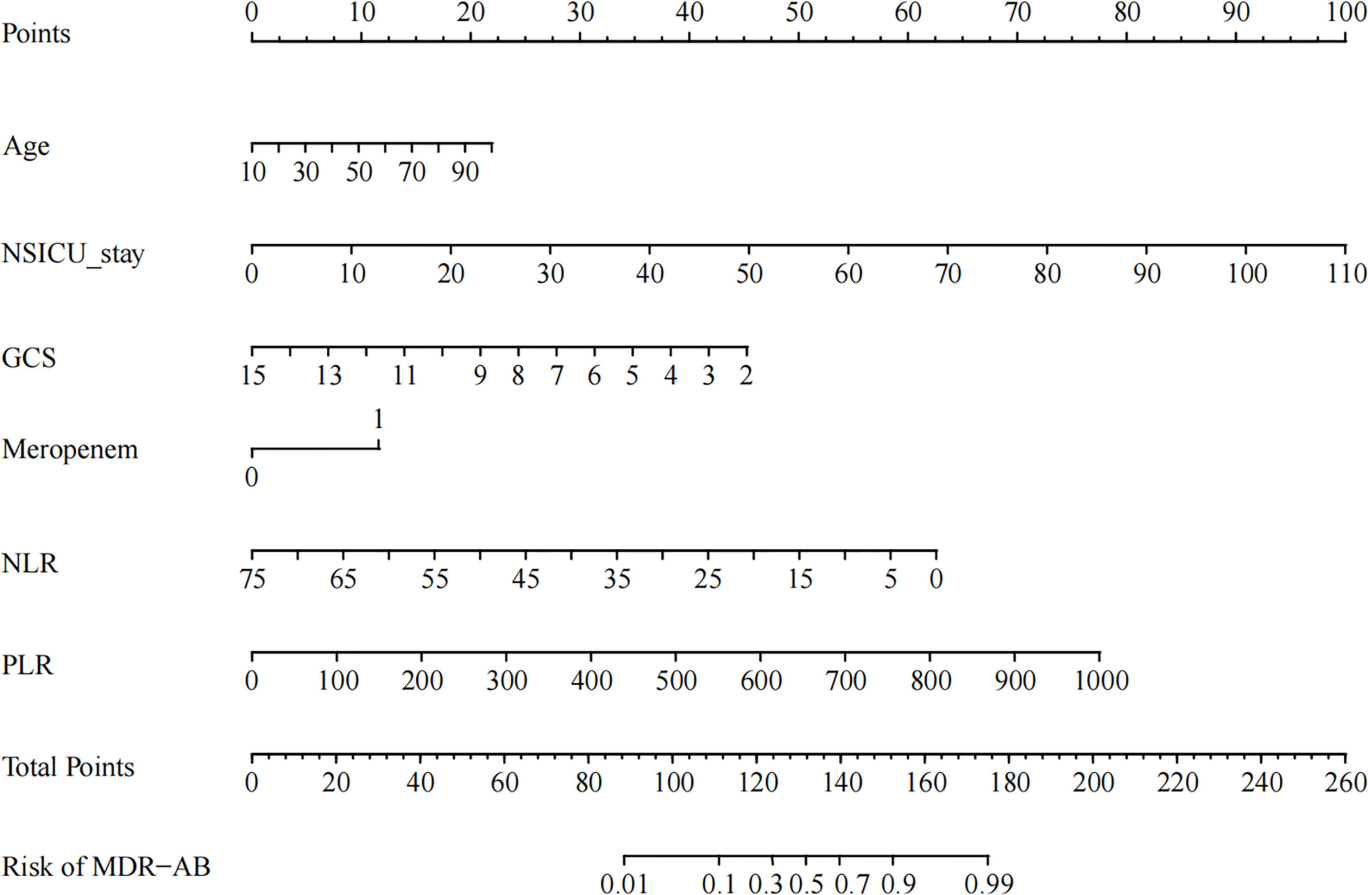
Example of prediction nomogram for the risk of pulmonary infection caused by multidrug-resistant *Acinetobacter Baumannii* (MDR-AB). *NLR*, neutrophil to lymphocyte ratio; *PLR*, platelet to lymphocyte ratio.

As shown in **Figure 2**, we constructed a nomogram based on the multivariate logistic regression model. For the values of each predictor in the nomogram, a vertical line is drawn upward from each point, and the values of different variables can correspond to different scores on the topmost reference line (scores range from 0 to 100). The scores corresponding to each predictor were summed to obtain the total score, and a vertical line was dropped from the total point line to the bottom probability line to determine the probability of pulmonary infection caused by MDR-AB.

**Figure 3:**
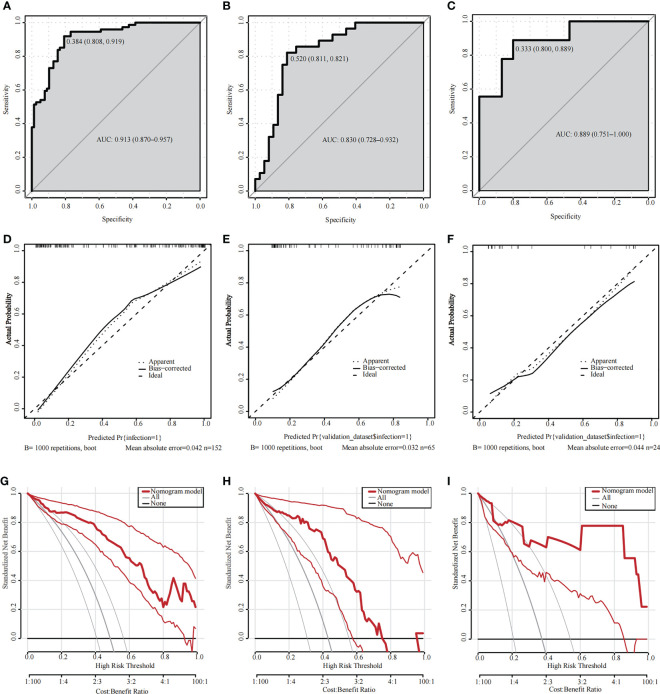
**(A–C)** ROC curves for validating the discrimination of the nomogram. **(A)** Primary cohort; **(B)** Validation cohort 1; **(C)** Validation cohort 2 (AUC=0.913 vs 0.830 vs 0.889). **(D–F)** Calibration curves for validating the calibration of the nomogram. **(D)** Primary cohort, HL test (χ^2 =^ 0.3673 and P value=0.8322); **(E)** Validation cohort 1, HL test (χ^2 =^ 0.2493 and P value=0.3801); **(F)** Validation cohort 2, HL test (χ^2 =^ 0.93241 and P value=0.6274). **(G–I)** DCA curves for validating the net income of the nomogram. **(G)** Primary cohort; **(H)** Validation cohort 1; **(I)** Validation cohort 2. ROC, receiver operating characteristic; AUC, area under the ROC curve; HL, Hosmer–Lemeshow; DCA, Decision curve analysis.

**Figures 3A, B** shows that the AUC of the nomogram was 0.913 (95% CI = 0.870–0.957) and 0.830 (95% CI = 0.728–0.932) for the primary cohort and validation cohort 1, respectively. In the primary cohort, the optimal cutoff for the nomogram was 0.384, with a sensitivity and specificity of 0.808 and 0.919, respectively. In validation cohort 1, the optimal cutoff was 0.520, sensitivity was 0.811, and specificity was 0.821. The above results show that our nomogram has good predictive ability.

**Figures 3D, E** shows that the calibration curves for the primary cohort and validation cohort 1 were similar to the 45-degree diagonal alignment. The HL test showed no significant difference (primary cohort: χ^2 =^ 0.3673, P=0.8322; validation cohort 1: χ^2 =^ 0.2493, P=0.3801), indicating that the nomogram had a satisfactory fit.

We then plotted a DCA curve to illustrate the value of the nomogram’s clinical application. **Figures 3G, H** shows that if the threshold probability is between 0.1 and 0.8 (in either cohort), the clinical intervention guided by the nomogram has a greater net benefit.

Finally, we prospectively collected data from 24 patients (MDR-AB pneumonia group, N=9; other bacterial pneumonia group, N=15) admitted to the NSICU as validation cohort 2 for external validation based on the six selected predictors (**Table 3**). The constructed nomogram had excellent discriminatory validity (**Figure 3C**, AUC=0.889), calibration validity (**Figure 3F**, HL test, P=0.6274), and clinical utility (**Figure 3I**, threshold probability, 0.1–1).

**Table 3:** Clinical characteristics of patients in the case and control groups in the validation cohort 2.

Predictor	Case group(N=9)	Control group (N=15)
Age (years)	72.1 ± 9.73	62.5 ± 19.6
NSICU stay (days)	26.0 (19.0,32.5)	13.0 (7.00,24.0)
GCS (points)	6.00 (3.00,9.00)	10.0 (9.0,12.0)
Meropenem	5 (55.6)	7 (46.7)
NLR	20.7 (9.79,25.2)	13.0 (6.90,16.6)
PLR	197.2 (127.4,581.1)	202.4 (116.7,242.1)

Data are shown as number (percentage), mean ± standard deviation, or median (interquartile range).

NSICU, neurosurgical intensive care unit; GCS, Glasgow Coma Scale; NLR, neutrophil to lymphocyte ratio; PLR, platelet to lymphocyte ratio.

## Discussion

In this study, conducted to identify novel risk factors of MDR-A-caused pulmonary infections in the NSICU and their associated effects, we found a very high incidence of pulmonary infections caused by MDR-AB in the NSICU and a high percentage of XDR-AB pneumonia among them. In addition to causing prolonged hospitalization and greatly increasing the economic burden on the patient’s family, the infections also cause complications and higher mortality rates (Zhen et al., 2021). Therefore, there is an urgent need for early detection and control of pulmonary infections caused by MDR-AB. Our study was based on the clinical data of NSICU patients, and the data were analyzed using an effective mathematical modeling approach. The final results indicated that older age, prolonged NSICU stay, lower GCS score, application of meropenem, lower NLR, and higher PLR were independent risk factors for pulmonary infection caused by MDR-AB. Subsequently, we developed a predictive nomogram consisting of these six predictors, with significantly high sensitivity and specificity for the early identification of infection (primary cohort AUC=0.913, validation cohort 1 AUC=0.830, validation cohort 2 AUC=0.889). DCA showed that the nomogram has great potential for clinical use. To the best of our knowledge, this is the first study to construct a nomogram for predicting the risk of pulmonary infection caused by MDR-AB.

To date, several studies have reported the risk factors for pulmonary infection due to single- and MDR-AB, all based on hospital-integrated ICU or pediatric ICUs (PICUs). Risk factors include the severity of the underlying disease, duration of hospital stay, duration of ICU stay, mechanical ventilation, invasive procedures, and prior antibiotic use (Munoz-Price and Weinstein, 2008; Cai et al., 2012; Zheng et al., 2013; Tsakiridou et al., 2014; Li et al., 2017; Ren et al., 2019). Some studies have established predictive models based on relevant clinical symptoms (such as anemia and hypoalbuminemia), clinical indicators (such as duration of hospitalization and ventilator usage), and biological indicators (such as LYM, NLR, and MLR), which can indicate the onset of pneumonia and even predict the prognosis of the patient (Seligman et al., 2006; Cataudella et al., 2017; Huang et al., 2018; Wu et al., 2021). Zhang et al. developed a nomogram from a single center that predicted the risk of death in patients with AB infection. This study included 10 factors from participants and did not involve laboratory indicators (Zhang et al., 2020). However, there are few studies on the early prediction of pulmonary infections caused by MDR-AB, and the research components are incomplete.

Most patients in the NSICU suffer from acute and critical diseases with relatively rapid progression and a high risk of deterioration. Neurosurgical disorders result in relatively poor consciousness and low physical activity in patients. In addition, it is challenging for many to be independent and have difficulty in actively cooperating with the clinical treatment. These factors contribute to the higher prevalence of AB in NSICUs (Ryu et al., 2017). Our study showed that older age significantly increased the risk of MDR-AB pneumonia. Compared with patients in other wards, the average age of patients admitted to the NSICU is higher, most have impaired consciousness including lethargy and coma, and they remain on bed rest for a longer period. Some patients also have symptoms, such as irritability and delirium, which cause difficulties in administering nursing care and treatment. However, in the primary cohort of this study, there was no statistically significant difference with respect to diseases such as hypertension, diabetes, coronary artery disease, and obsolete cerebral infarction. This suggests that the patient’s diagnosis and underlying disease at admission were not the primary cause of MDR-AB pulmonary infection, relative to patients with pulmonary infections caused by other bacteria. Moreover, it substantiates that case and control groups included in this study are comparable and that the experimental results are more authentic.

The GCS is an accessible physiological scoring system that can assess the degree of impaired consciousness in patients on admission (Alvarez et al., 1998). In our study, GCS was identified as a predictor of pulmonary infection caused by MDR-AB, and the risk of infection decreased with increasing GCS scores. AB is a conditionally pathogenic bacterium present in the respiratory and gastrointestinal tracts. A low GCS score indicates that the patient is in a poor state of consciousness, and brainstem reflexes may be delayed or absent. This can greatly prolong the patient’s time remaining bedridden, which in turn reduces the patient’s ability to expel sputum and ventilate, causing AB from the respiratory and gastrointestinal tracts to infect the lungs (Chen et al., 2013). With a prolonged stay in the NSICU, the immunity of elderly patients is reduced. If there are no effective nursing measures, such as turning over and expelling sputum in time, it could lead to hypostatic pneumonia (Ji et al., 2022).

Many studies have suggested that longer hospital stays can increase the risk of pulmonary infections in patients with AB (Munoz-Price and Weinstein, 2008). We did not confirm this notion in this study; however, we only found that pulmonary infection caused by MDR-AB was associated with a prolonged stay in the NSICU. This could be due to the fact that the infection exacerbates neurological symptoms and leaves the patient in a state of prolonged unconsciousness. The modes of transmission of AB include contact (direct and indirect), droplet, and airborne modes, and prolonged exposure of patients to the NSICU, which can significantly increase the chance of infection. Elderly patients admitted to the NSICU are at an increased risk of infection, as their immunity decreases throughout the disease course (Yakupogullari et al., 2016). Patients are transferred back to the general neurosurgery ward for further treatment after their condition improves. The degree of bacterial resistance and invasiveness in general wards is lower than that in ICUs, which can effectively prevent the occurrence of MDR-AB pulmonary infections (Donowitz et al., 1982).

The resistance mechanisms of AB to carbapenem antibiotics include the production of carbapenemase to hydrolyze the antibiotic drug, reduced affinity for penicillin-binding proteins, downregulation of outer membrane protein expression, and active excretion of the drug from the bacteria (Duraes et al., 2018). Related studies have shown that the use of carbapenems is a risk factor for infection with AB pneumonia (Falagas and Kopterides, 2006). Carbapenems include imipenem, meropenem, and biapenem; however, our study showed that the application of meropenem increased the risk of infection with MDR-AB pneumonia. This may be due to the low use of both imipenem and biexpenem in the departments where we conducted our study, making the amount of clinical data too small to be statistically meaningful. Neurosurgery is one of the most challenging and delicate procedures among all surgical systems. Most of the patients admitted are of advanced age with underlying diseases and are immunocompromised, with high susceptibility to postoperative intracranial infections (Xiao et al., 2022). Owing to the low success rate of bacterial cultures in cerebrospinal fluid (approximately 20%) and the high mortality rate of intracranial infections, meropenem is commonly used in the treatment of empirical central infections in post-neurosurgical patients, and its application rate in the clinic is increasing (Tunkel et al., 2017; Schneider et al., 2022). Our study found that meropenem application increased the risk of MDR-AB pneumonia, whereas all generations of cephalosporins and vancomycin were not associated with an increased risk. The use of meropenem should be controlled in clinical practice. For surgical patients, we can apply first or second-generation cephalosporins (*cefuroxime, cefoxitin*) for infection prevention in the perioperative period, and the course of treatment usually does not exceed 24 hours. The patient may be treated empirically with a third or fourth-generation cephalosporin (*ceftriaxone, cefepime*) instead of meropenem and combined with vancomycin first if the possibility of intracranial infection complications is considered. This allows for full coverage against both gram-positive and gram-negative bacteria. If the treatment is ineffective or after the sensitive antibiotic for the bacteria is confirmed by cerebrospinal fluid culture, meropenem can be replaced or added. In addition, empirical anti-infective therapy should be administered with cephalosporins or carbapenems, as appropriate, considering local epidemiologic data, as well as the distribution and resistance of pathogenic bacteria.

Inflammatory indicators such as NLR, LMR, and PLR, whose reliability and accessibility are established in almost all medical disciplines, can be used as markers of the immune response to various infectious and non-infectious stimuli (Russell et al., 2019). In this study, early NLR and PLR were screened as independent risk factors by stepwise regression and included in our nomogram. Although NLR was ultimately identified as a protective factor, it acted in conjunction with PLR in this nomogram to affect the final probability of infection.

The age and GCS score of the patient cannot be controlled by physicians. Relevant security authorities such as senior care facilities and traffic police can improve their measures and efficiency, focus on elderly patients, prevent the occurrence of traumatic brain injury, and reduce the severity of its symptoms. It is important for physicians to control the duration of hospitalization, standardize the use of meropenem, and pay attention to early inflammatory indicators to reduce the incidence of MDR-AB pulmonary infection.

This study has several strengths. Firstly, our study provides a convenient and practical quantitative prediction tool based on only six predictors that are readily available from routine clinical and blood tests. Secondly, the inclusion and exclusion criteria were rigorously developed for this study, and sufficient study subjects and data on factors were collected. Finally, our nomogram was validated extensively and showed good discrimination, calibration, and robust clinical utility and stability.

This study has some limitations: Firstly, this study retrospectively documented patient information from a single center for 2 years and did not use external data for validation. A multicenter study with a larger sample size is needed to validate the results of this study. Secondly, the predictive model we proposed was based on the NSICU of Tianjin Medical University General Hospital. This is an exploration of a quantitative model that may not be widely applicable to various disciplines, and the coefficients of the relevant factors in the model may vary from case to case.

In conclusion, our nomogram incorporated patient age, duration of NSICU stay, GCS, early NLR, and PLR. The occurrence of pulmonary infections caused by MDR-AB can be predicted at the early stage of hospitalization. According to the predicted results, it is possible to advise patients with sputum bacterial culture and drug susceptibility testing at an early stage. Switching from empirical treatment to targeted treatment before the diagnosis of MDR-AB pulmonary infection can improve patients’ recovery and survival rates. Overall, our study will help in early prediction and implementation of targeted interventions against pulmonary infection caused by MDR-AB for better patient management.

## Data availability statement

The raw data supporting the conclusions of this article will be made available by the authors, without undue reservation.

## Ethics statement

The study was approved by the Ethics Committee of Tianjin Medical University General Hospital. Owing to the retrospective nature of this study, there was no interference in the patients’ diagnosis and treatment, although a small amount included some prospectively collected clinical information of the patients for validation; therefore, obtaining informed consent from the patients was waived by the ethics committee. This study complied with the Declaration of Helsinki and adhered to the principles of medical ethics. The authors kept all patient information strictly confidential.

## Author contributions

RJ and DW contributed substantially to the collection of literature, study design, data interpretation, and manuscript writing. RJ, JS, JL, and CP reviewed and revised the articles and performed the quality assessment. WJ, ML, MN, CW, TL, YC, JF, and SD performed data collection and statistical analyses. ZS, YF, and JY interpreted the data and studied the oversight. All authors contributed to the article and approved the submitted version.
