# Supplementary material for: Evaluating the efficiency of a nomogram based on the data of neurosurgical intensive care unit patients to predict pulmonary infection of multidrug-resistant Acinetobacter baumannii
Source: Front Cell Infect Microbiol. 2023 Apr 25;13:1152512. doi: 10.3389/fcimb.2023.1152512 (PMC10167012; doi:10.3389/fcimb.2023.1152512)
Supplement: Supplementary file 1 [file Table_1.docx]

| **Supplementary table S1 Patient characteristics of the case and control groups** | | | |
| --- | --- | --- | --- |
| Variables | Case group (N=102) | Control group (N=115) | P-value |
| **On admission** | | | |
| Gender (males) | 67(65.7) | 84(73.0) | 0.240 |
| Age (years) | 61.7±16.0 | 60.8±14.7 | 0.661 |
| Primary diagnosis |  |  |  |
| Cerebral hemorrhage | 54(52.9) | 58(50.4) | 0.712 |
| Traumatic brain injury | 40(39.2) | 27(23.5) | 0.012 |
| Intracranial space occupying lesion | 3(2.94) | 8(6.96) | 0.178 |
| Intracranial infections | 4(3.92) | 1(0.87) | 0.135 |
| Ischemic cerebrovascular disease | 5(4.90) | 11(9.57) | 0.190 |
| Aneurysm | 2(1.96) | 12(10.4) | 0.011 |
| Others | 2(1.96) | 5(4.35) | 0.451 |
| Comorbidities |  |  |  |
| Rib fractures | 13(12.7) | 7(6.09) | 0.091 |
| Pulmonary contusion | 8(7.84) | 6(5.22) | 0.432 |
| Systemic multiple fractures | 17(16.7) | 7(6.09) | 0.013 |
| Hypertension | 55(53.9) | 72(62.6) | 0.195 |
| Diabetes | 24(23.5) | 29(25.2) | 0.773 |
| Hyperlipidemia | 6(5.88) | 8(6.96) | 0.748 |
| Obsolete cerebral infarction | 24(23.5) | 23(20.0) | 0.529 |
| Obsolete cerebral hemorrhage | 12(11.8) | 8(6.96) | 0.222 |
| Atrial fibrillation | 3(2.94) | 5(4.35) | 0.725 |
| Coronary heart disease | 12(11.8) | 17(14.8) | 0.514 |
| **After admission** |  |  |  |
| Total duration of hospitalization (days) | 47.0(30.0,72.0) | 30.0(21.0,46.0) | <0.001 |
| NSICU stay (days) | 29.5(19.0,43.3) | 14.0(9.00,20.0) | <0.001 |
| GCS (points) | 6.50(4.00,8.25) | 10.0(8.00,13.0) | <0.001 |
| Pressure ulcer risk assessment score | 12.0(11.0,13.0) | 12.0(11.0,13.0) | 0.188 |
| APACHE Ⅱ score (points) |  |  | <0.001 |
| 10- <15 | 26(25.8) | 54(47.1) |  |
| 15- <25 | 60(59.1) | 53(46.2) |  |
| ≥25 | 16(15.1) | 8(6.67) |  |
| Ventilator usage time (hours) |  |  | <0.001 |
| <24 | 27(26.9) | 62(62.9) |  |
| 24- <96 | 16(14.0) | 19(16.2) |  |
| ≥96 | 59(59.1) | 24(21.0) |  |
| Tracheal intubation | 67(65.7) | 71(61.7) | 0.546 |
| Tracheotomy | 33(32.4) | 22(19.1) | 0.025 |
| Electronic bronchoscopy | 33(32.4) | 12(10.4) | <0.001 |
| Urinary catheterization | 97(95.1) | 112(97.4) | 0.371 |
| Gastric tube placement | 93(91.2) | 101(87.8) | 0.424 |
| Tertiary /quaternary surgery | 50(49.0) | 67(58.3) | 0.173 |
| Lumbar puncture | 36(35.3) | 23(20.0) | 0.011 |
| Limb motor ability | 48(47.1) | 66(57.4 ) | 0.128 |
| Protective restraint | 47(46.1) | 58(50.4) | 0.522 |
| **Antibiotic applications** |  |  |  |
| Cefazolin | 6(5.88) | 3(2.61) | 0.311 |
| Cefoxitin | 42(41.2) | 59(51.3) | 0.135 |
| Cefuroxime | 2(1.96) | 7(6.09) | 0.128 |
| Ceftriaxone | 10(9.80) | 10(8.70) | 0.778 |
| Ceftazidime | 3(2.94) | 2(1.74) | 0.668 |
| Vancomycin | 26(25.5) | 26(22.6) | 0.620 |
| Linezolid | 13(12.7) | 7(6.09) | 0.091 |
| Imipenem | 12(11.8) | 12(10.4) | 0.755 |
| Meropenem | 53(52.0) | 27(23.5) | <0.001 |
| Biapenem | 2(1.96) | 1(0.87) | 0.602 |
| Piperacillin-tazobactam | 62(60.8) | 74(64.3) | 0.588 |
| Cefoperazone-sulbactam | 28(27.5) | 48(41.7) | 0.028 |
| Teicoplanin | 5(4.90) | 7(6.09) | 0.703 |
| Levofloxacin | 2(1.96) | 8(6.96) | 0.108 |
| **Laboratory test** |  |  |  |
| WBC (×10^9^/L) | 9.86(8.50,11.9) | 10.3(8.42,12.1) | 0.760 |
| PLT (×10^9^/L) | 161.5(121.9,194.0) | 164.0(140.0,199.0) | 0.128 |
| NEU (×10^9^/L) | 8.24(6.87,10.2) | 8.67(6.75,10.3) | 0.828 |
| LYM (×10^9^/L) | 0.66(0.50,0.91) | 0.84(0.60,1.12) | 0.001 |
| MON (×10^9^/L) | 0.74(0.55,0.99) | 0.76(0.59,0.96) | 0.775 |
| HGB (g/L) | 101.5(89.8,119.6) | 115.5(99.5,129.0) | 0.001 |
| GLO (g/L) | 29.5(26.0,32.3) | 29.0(26.5,31.5) | 0.409 |
| ALB (g/L) | 31.0(28.5,34.0) | 32.0(29.5,34.5) | 0.046 |
| ALT (U/L) | 31.5(18,0,66.0) | 29.5(16.5,47.5) | 0.059 |
| AST (U/L) | 44.5(30.4,69.5) | 32.5(25.0,54.5) | 0.001 |
| CREA (umol/L) | 58.0(46.5,77.4) | 57.0(46.0,69.5) | 0.408 |
| NLR | 12.7(9.06,17.9) | 8.95(7.00,16.4) | 0.004 |
| LMR | 0.91(0.64,1.37) | 1.09 (0.81,1.41) | 0.024 |
| PLR | 234.2(180.9,302.0) | 194.2(149.4,274.7) | 0.027 |
| CRP (mg/dL) | 7.34(3.84,11.6) | 7.12(3.51,10.8) | 0.789 |
| PCT (ng/mL) | 0.36(0.09,0.87) | 0.22(0.05,0.71) | 0.139 |
| D-Dimer(ng/mL) | 2591.0(1154.0,5117.0) | 1566.0(800.0,3480.5) | 0.016 |
| GLU(umol/L) | 8.30(6.68,10.1) | 7.8(6.25,10.0) | 0.214 |
| Data are shown as number (percentage), mean±standard deviation, or median (interquartile range).  Abbreviations: NSICU, neurosurgical intensive care unit; GCS, Glasgow Coma Scale; WBC, white blood cell count; PLT, platelets count; NEU, absolute neutrophil count; LYM, absolute lymphocyte count; MON, absolute monocyte count; HGB, hemoglobin; GLO, globulin; ALB, albumin; ALT, alanine transaminase; AST, aspartate transaminase; NLR, neutrophil to lymphocyte ratio; LMR, lymphocyte to monocyte ratio; PLR, platelet to lymphocyte ratio; CRP, C-reactive protein; PCT, descendants; GLU, glucose. | | | |
